# Ceramide accumulation induces mitophagy and impairs β-oxidation in PINK1 deficiency

**DOI:** 10.1073/pnas.2025347118

**Published:** 2021-10-22

**Authors:** Melissa Vos, Marija Dulovic-Mahlow, Frida Mandik, Lisa Frese, Yuliia Kanana, Sokhna Haissatou Diaw, Julia Depperschmidt, Claudia Böhm, Jonas Rohr, Thora Lohnau, Inke R. König, Christine Klein

**Affiliations:** ^a^Institute of Neurogenetics, University of Luebeck, 23562 Luebeck, Germany;; ^b^Institut für Medizinische Biometrie und Statistik, University of Luebeck, 23562 Luebeck, Germany

**Keywords:** Parkinson’s disease, PINK1, mitochondria, ceramide, β-oxidation

## Abstract

Energy production via the mitochondrial electron transport chain (ETC) and mitophagy are two important processes affected in Parkinson’s disease (PD). Interestingly, PINK1, mutations of which cause early-onset PD, plays a key role in both processes, suggesting that these two mechanisms are connected. However, the converging link of both pathways currently remains enigmatic. Recent findings demonstrated that lipid aggregation, along with defective mitochondria, is present in postmortem brains of PD patients. In addition, an increasing body of evidence shows that sphingolipids, including ceramide, are altered in PD, supporting the importance of lipids in the pathophysiology of PD. Here, we identified ceramide to play a crucial role in PINK1-related PD that was previously linked almost exclusively to mitochondrial dysfunction. We found ceramide to accumulate in mitochondria and to negatively affect mitochondrial function, most notably the ETC. Lowering ceramide levels improved mitochondrial phenotypes in *pink1*-mutant flies and PINK1-deficient patient-derived fibroblasts, showing that the effects of ceramide are evolutionarily conserved. In addition, ceramide accumulation provoked ceramide-induced mitophagy upon PINK1 deficiency. As a result of the ceramide accumulation, β-oxidation in *PINK1* mutants was decreased, which was rescued by lowering ceramide levels. Furthermore, stimulation of β-oxidation was sufficient to rescue PINK1-deficient phenotypes. In conclusion, we discovered a cellular mechanism resulting from PD-causing loss of PINK1 and found a protective role of β-oxidation in ETC dysfunction, thus linking lipids and mitochondria in the pathophysiology of PINK1-related PD. Furthermore, our data nominate β-oxidation and ceramide as therapeutic targets for PD.

## Significance

Ceramide accumulates in Parkinson’s disease–related PINK1 deficiency to initiate ceramide-mediated mitophagy as an alternative pathway to overcome defective PINK1-related mitophagy and the concomitant increased requirements for mitochondrial clearance. Increased ceramide levels negatively correlate with β-oxidation and thus decrease efficiency of the electron transport chain, further increasing the need for mitochondrial clearance. Interfering with this vicious cycle can constitute a novel therapeutic strategy as suggested by our data showing that a reduction of ceramide levels or stimulation of β-oxidation improve the *PINK1*-mutant phenotypes.

Loss of PINK1 function causes autosomal recessive early-onset Parkinson’s disease (PD). Most patients present with bradykinesia, rigidity, resting tremor, and dyskinesia and are responsive to dopamine replacement therapy (1). On the cellular level, *PINK1* disease mutations result in impaired energy metabolism and a variety of mitochondrial defects that can partially be alleviated by stimulation of energy metabolism (2–4). Intriguingly, abnormal mitochondrial morphology, along with lipid aggregates, was recently discovered to be present in Lewy bodies of postmortem PD patients’ brains (5), challenging the previously held notion of alpha-synuclein being the almost exclusive neuropathological correlate. This finding confirms the involvement of mitochondrial dysfunction in PD and additionally suggests a critical role of lipids in the pathogenesis of PD.

PINK1 is important for the phosphorylation of the Complex I subunit NdufA10 resulting in efficient Complex I and electron transport chain (ETC) activity (6, 7). This function is evolutionarily conserved between *Drosophila* and humans. Hence, in both flies and humans, loss of PINK1 results in an impaired ETC, reduced ATP levels, and defective mitochondrial morphology (6, 8, 9), all of which are ubiquitously observed in the fly already at the early larval stage. Furthermore, alongside Parkin, PINK1 plays a crucial role in mitophagy to remove defective mitochondria that appears to be defective in an age-dependent fashion (10–13). *Pink1*-mutant *Drosophila melanogaster* additionally show thorax muscle degeneration and defective flying ability (8, 9). These latter defects, together with impaired mitochondrial morphology, can be rescued by expressing the fission-promoting protein Drp1 (14). However, increased fission does not improve ETC-related defects (15). Furthermore, stimulation or facilitation of the ETC rescues ETC-related phenotypes in *pink1*-mutant *Drosophila*, including ATP levels and mitochondrial morphology (3, 4, 7, 15, 16). These data collectively suggest two parallel mechanisms that converge on a shared common pathway leading to the development of PD. However, the link between these two pathways has yet to be resolved.

Recently, disrupted lipid homeostasis has garnered increasing attention in PD (16–18). Furthermore, ceramide, the basic sphingolipid, is altered in several PD models and has been implicated in PD-related alpha-synuclein toxicity (17–20). Interestingly, ceramide induces mitophagy that is facilitated by Drp1 (21). Furthermore, pathogenic variants in Glucocerebrosidase (GCase), an enzyme involved in ceramide synthesis, are known to be the most common risk factor for PD (22, 23). However, the exact mechanism remains enigmatic. We found increased ceramide levels in isolated mitochondria of *Pink1^−/−^* knockout (KO) mouse embryonic fibroblasts (MEFs) (16) and Pink1-deficient flies. Increased ceramide levels are detrimental for proper ETC function (24). Hence, we hypothesize that ceramide accumulation in PINK1 deficiency affects ETC function and mitophagy and constitutes the missing link between these two important processes affected in PD.

## Results

### Genetic Inhibition of Ceramide Synthase Rescues Mitochondrial Phenotypes in Pink1-Deficient Flies.

**Fig. 1.**
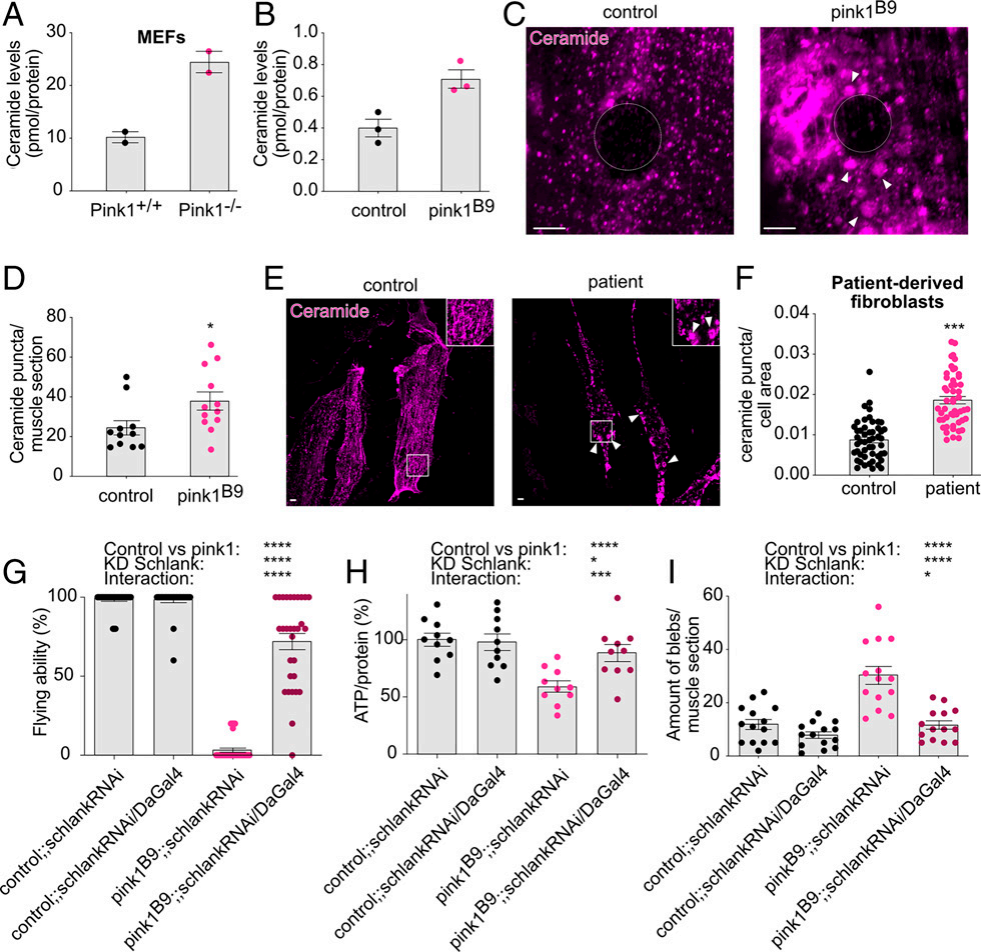
Genetic inhibition of ceramide synthase rescues mitochondrial phenotypes in Pink1-deficient flies. (*A* and *B*) Ceramide levels in isolated mitochondria from *Pink1^−/−^* KO MEFs (*n* = 2) (*A*) and in *pink1^B9^*-mutant flies (*n* = 3) (*B*) compared to controls. (*C*–*E*) Ceramide forms accumulations (ceramide puncta ≥ 0.5 μm^2^) in Pink1-deficient flies compared to control flies (*n* = 11 to 12 muscle sections; 4 larvae) (*P* < 0.05) (*C*: images and *D*: quantification; quantification full intensity [*SI Appendix*, Fig. S1*A*]) and in patient-derived fibroblasts carrying homozygous *PINK1* mutations compared to fibroblasts from age-matched controls (*n* = 25 cells per cell line [two control cell lines and two patient cell lines]) (*P* < 0.001) (*E*: images and *F*: quantification). (*G*–*I*) Knockdown of *drosophila* ceramide synthase (Schlank) in *pink1^B9^*-mutant flies showed increased flying ability (*n* > 50 flies) (*P* [pink1 versus control] < 0.0001; *P* [DaGal4] < 0.0001; *P* [interaction] < 0.0001); of note, we do not compare the groups in a univariate setting (i.e., test whether the shown four groups are different), followed by pairwise comparisons. Instead, the factorial design of the experiments is taken into account. One factor is the group (control versus pink1), the other is the knockdown (yes versus no). We can therefore test three effects, namely, 1) if the group has an effect, independent of the knockdown, 2) if the knockdown has an effect independent of the group, and 3) if there is an interaction between these two factors (i.e., if the effect of the knockdown depends on the group). (*G*) ATP levels (*n* = 10 assays of 2 flies/assay) (*P* [pink1 versus control] < 0.0001; *P* [DaGal4] < 0.05; *P* [interaction] < 0.001) and (*H*) improved mitochondrial morphology that is indicated by a reduction of the amount of mitochondrial accumulations (= blebs ≥ 0.25 μm^2^) (*n* = 14 muscle sections; 4 larvae) (*P* [pink1 versus control] < 0.0001; *P* [DaGal4] < 0.0001; *P* [interaction] < 0.05) (*I*: quantification and *SI Appendix*, Fig. S1*F*: images). Panels that do not present data from flies are labeled accordingly. Data are individual data points; bars are means (*A* and *B*; *D*; *F* and *I*) or percentages (*G* and *H*); error bars: SEM (*D*); (*F*) Mann–Whitney *U* test; (*G*–*I*) nonparametric ANOVA; *P < 0.05; ***P < 0.001; ****P < 0.0001; pairwise comparisons as guidance can be found in the supplemental information (*G*–*I*); circles depict the cell nucleus; arrowheads indicate the accumulations (ceramide and mitochondria). (Scale bar, 5 μm).

In a previously performed lipidomic analysis (16), we observed an accumulation of ceramide in isolated mitochondria of *Pink1^−/−^* MEFs compared to mitochondria isolated from wild-type MEFs (Fig. 1*A*). Lipidomic analyses of isolated mitochondria of *pink1*-mutant adult flies similarly point to increased ceramide levels compared to controls (Fig. 1*B*), confirming the initial data in MEFs (Fig. 1*A*). In addition, ceramide immunolabeling revealed an increased number of accumulations of ceramide in larval muscle and in adult brain tissue (Fig. 1 *C* and *D* and *SI Appendix*, Fig. S1 *A* and *B*) compared to controls, further supporting the observation of altered ceramide distribution in *pink1*-mutant flies. Next, we assessed the ceramide distribution in patient-derived fibroblasts endogenously carrying homozygous *PINK1* mutations using two independent cultures (25) along with age-matched controls and pooled the data for patients and controls, respectively. Ceramide labeling in patient-derived fibroblasts showed an altered distribution of ceramide indicated by the increased puncta compared to control fibroblasts (Fig. 1 *E* and *F*), suggesting the defective ceramide homeostasis upon PINK1 deficiency is evolutionarily conserved. Intensity levels of ceramide in *pink1*-mutant flies are higher compared to control flies (*SI Appendix*, Fig. S1*C*), which is further supported by our lipidomic analyses of ceramide in adult flies revealing increased ceramide levels (*SI Appendix*, Fig. S1*D*). While accumulation of ceramide labeling is different in patient-derived fibroblasts compared to control fibroblasts (Fig. 1 *E* and *F*), intensity analyses did not show a difference as a consequence of loss of PINK1 (*SI Appendix*, Fig. S1*E*). Overall, these findings underline the importance of the altered distribution of ceramide as a result of PINK1 deficiency.

**Fig. 2.**
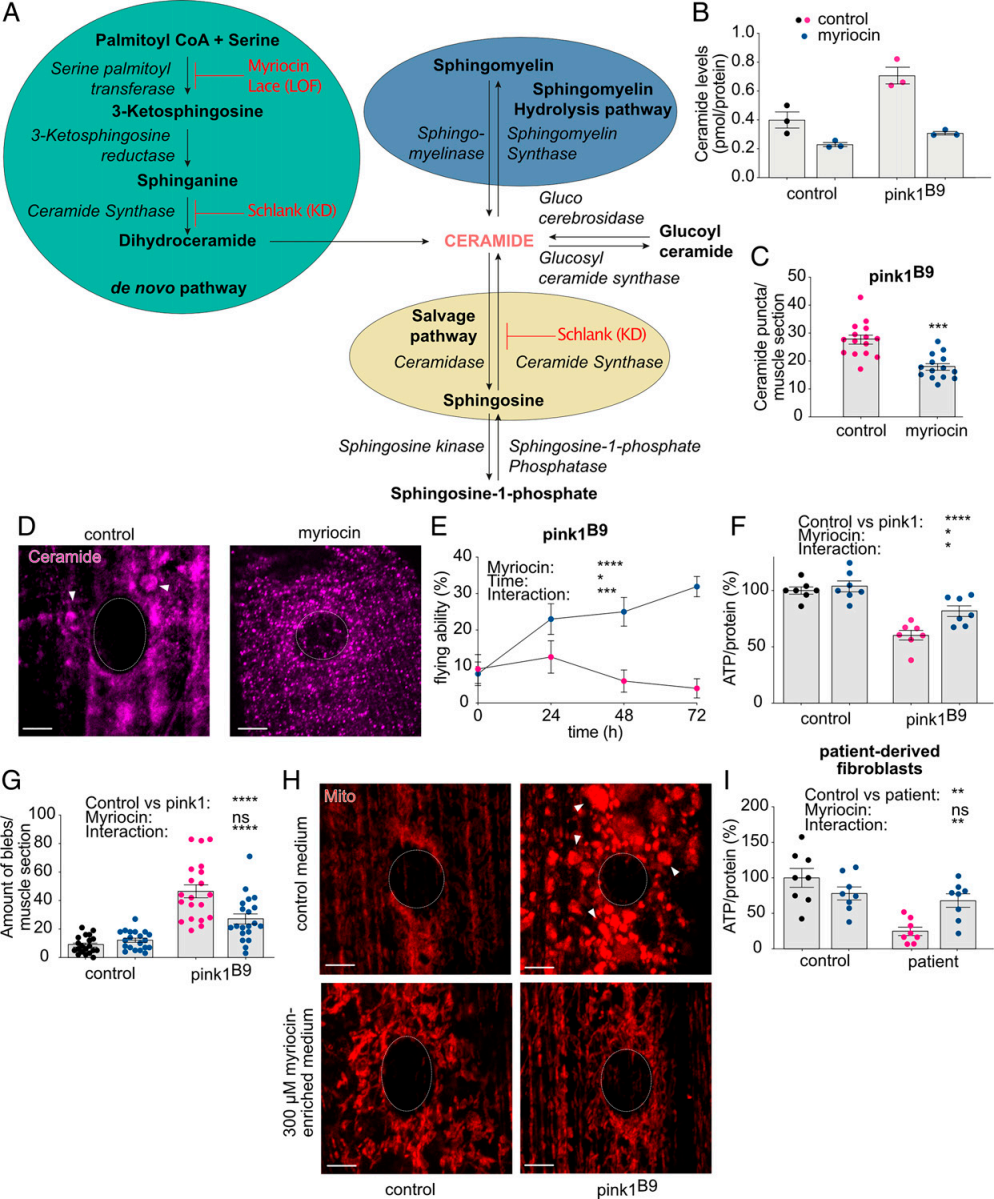
Pharmacological inhibition of ceramide synthesis rescues Pink1 deficiency. (*A*) Scheme of the different pathways of ceramide synthesis, including steps that were blocked genetically and pharmacologically in the present study. (*B*) Ceramide levels following a 300 μM myriocin treatment for 72 h (blue dots) in isolated mitochondria of control and *pink1*-mutant flies (*n* = 3). (*C* and *D*) Ceramide accumulations (ceramide puncta ≥ 0.5 μm^2^) in Pink1-deficient flies are reduced upon treatment with 300 μM myriocin (blue dots) (*n* = 14 to 15 muscle sections; four larvae) (*P* < 0.001) (*C*: quantification and *D*: images). (*E*) Flying ability of *pink1^B9^*-mutant flies that were placed on 300 μM myriocin-enriched medium (blue dots) that were tested every 24 h (*n* > 50 flies) (*P* [myriocin] < 0.0001, *P* [time] < 0.05, and *P* [interaction] < 0.001]. (*F*–*H*) Supplementation of 300 μM myriocin for 72 h (blue dots) to the fly medium resulted in increased ATP levels (*n* = 7 assays of 2 flies/assay) (*P* [pink1 versus control] < 0.0001; *P* [myriocin] < 0.05; *P* [interaction] < 0.05) (*F*), improved mitochondrial morphology shown by the reduction of mitochondrial accumulations (blebs ≥ 0.25 μm^2^) (*n* = 20 muscle sections; 4 larvae) (*P* [pink1 versus control] < 0.0001; *P* [myriocin] = 0.0595; *P* [interaction] < 0.0001) (*G*: quantification; *H*: images). (*I*) Patient-derived fibroblasts carrying homozygous *PINK1* mutations show increased ATP levels following a 24 h treatment with 2.5 μM myriocin (blue dots) (*n* = 8 assays) (*P* [patient versus control] < 0.01, *P* [myriocin] = 0.0808, and *P* [interaction] < 0.01). Panels that do not present data from flies or that show data derived from *pink1*-mutant flies with and without drugs are labeled accordingly. Data are individual data points with means (*B* and *C*; *G*) or percentages (*E* and *F*; *I*); error bars: SEM. (*C*) Mann–Whitney *U* test; (*E*–*G*; *I*) nonparametric analyses of variance; ns: not significant; *P < 0.05; **P < 0.01; ***P < 0.001; ****P < 0.0001; pairwise comparisons as guidance can be found in the supplemental information (*F* and *G*; *I*); circles depict the cell nucleus; arrowheads indicate the accumulations (ceramide and mitochondria). (Scale bar, 5 μm.)

In mammalian cells, several ceramide synthases (CerS) exist to produce ceramide (26). Flies only have one CerS, termed Schlank. Thus, to test if the accumulation of ceramide contributes to the observed *pink1*-mutant phenotypes, we ubiquitously knocked down *schlank* that was previously shown to reduce ceramide levels (27) in Pink1-deficient flies using available ribonucleic acid interference (RNAi) lines. Knockdown of *schlank* in Pink1-deficient flies resulted in improved phenotypes, including flying ability, ATP levels, and mitochondrial morphology, while no effect was observed when knocked down in wild-type control flies (Fig. 1 *G*–*I* and *SI Appendix*, Fig. S1 *F*–*H*). Furthermore, heterozygous loss of Lace induced improved flying ability and ATP levels (*SI Appendix*, Fig. S1 *I* and *J*). Lace is the fly ortholog of serine palmitoyl transferase (SPT), which is the first enzymatic step of the de novo ceramide synthetic pathway (Fig. 2*A*). Hence, lowering ceramide via partial loss of ceramide synthetic enzymes rescues Pink1-deficient phenotypes. Interestingly, overexpression of Schlank to increase ceramide synthesis resulted in phenotypes that are in part similar to those observed upon loss of Pink1; namely, these flies have flying difficulties and abnormal mitochondrial morphology, whereas their ATP levels were not affected (*SI Appendix*, Fig. S1 *K*–*N*). This suggests that the observed ceramide accumulation in PINK1 deficiency plays a significant role in the *pink1*-mutant phenotypes.

### Pharmacological Inhibition of Ceramide Synthesis Rescues Pink1 Deficiency.

To confirm that our observations are ceramide-specific and the result of a reduction of ceramide levels, we supplemented the fly medium with myriocin, a potent inhibitor of SPT (Fig. 2*A*) (28). Myriocin effectively reduces ceramide levels in vitro and in vivo in different animal models (29, 30), which was further supported by our lipidomic analyses in which ceramide levels are reduced following treatment of 300 μM myriocin in isolated mitochondria (Fig. 2*B*). In addition, immunolabeling experiments showed that myriocin supplementation to the fly medium efficiently lowered the ceramide accumulations to normal levels (Fig. 2 *C* and *D*). We supplemented 300 μM to the fly medium, and the subsequent reduction of ceramide resulted in a time-dependent improvement of the *pink1^B9^-*mutant flying ability, increased ATP levels, and improved mitochondrial morphology of *pink1^B9^* mutants (Fig. 2 *E*–*H*). Inhibition of ceramide synthesis via myriocin did not have an effect on control flies (Fig. 2 *E*–*H*), which is similar to what we observed upon Schlank knockdown (Fig. 1 *G*–*I*). Thus, the observed rescue of *pink1-*mutant phenotypes is specifically due to reduced ceramide levels.

To test if these observations in flies are conserved in human cells, we employed 2.5 μM of myriocin for 24 h to patient-derived fibroblasts (25) to reduce ceramide levels. Previously, 1 μM of myriocin treatment was shown to reduce ceramide levels effectively to less than 60% (31). *PINK1*-mutant fibroblasts that were treated with myriocin showed increased ATP levels compared to those that were treated with control medium (Fig. 2*I*). Thus, the rescuing effect of reduction of ceramide upon PINK1 deficiency is evolutionarily conserved. Loss of Pink1 exhibits similar phenotypes to loss of Parkin, and it was previously shown that PINK1 and Parkin function together in mitophagy (32). In addition, previous studies have found altered levels of ceramide in *Parkin* KO mice models (18). Hence, we tested if the reduction of ceramide has a beneficial effect on Parkin deficiency too. *Park^1/Δ21^*-mutant flies were placed on 300 μM myriocin-enriched fly medium, and myriocin led to improved flying ability, increased ATP levels, and improved mitochondrial morphology (*SI Appendix*, Fig. S2 *A*–*D*), suggesting that ceramide is a shared player in PINK1- and Parkin-related PD.

### β-oxidation Is Decreased upon Loss of PINK1 Due to Ceramide Accumulation.

**Fig. 3.**
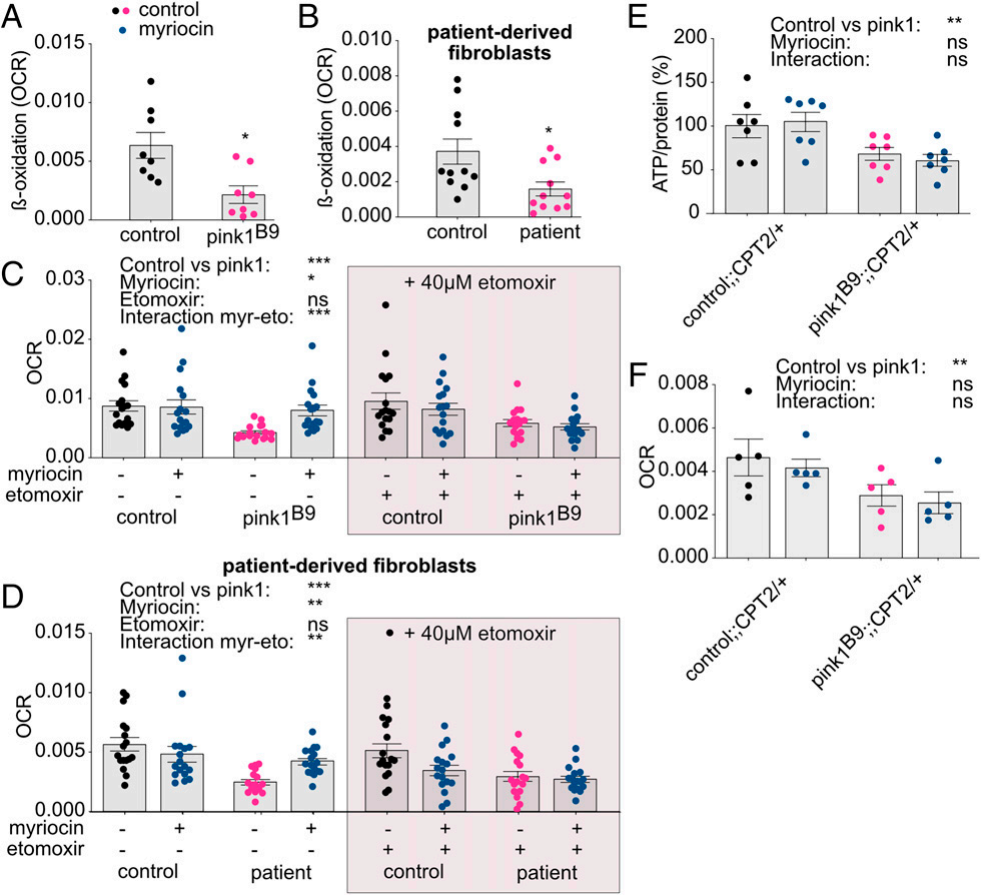
β-oxidation is decreased upon loss of PINK1 due to ceramide accumulation. (*A* and *B*) β-oxidation levels displayed as OCR is lowered in *pink1^B9^*-mutant flies (*n* = 8 assays) (*P* < 0.05) (*A*) and patient-derived fibroblasts (*n* = 11 assays) (*P* < 0.05) (*B*) compared to controls. (*C* and *D*) The reduced OCR upon PINK1 deficiency is rescued following 300 μM myriocin treatment for 72 h (blue dots) in flies (*n* = 17 assays) (*P* [pink1 versus control] < 0.001; *P* [myriocin] < 0.05; *P* [etomoxir] = 0.848; *P* [interaction: myriocin-etomoxir] < 0.001) (*C*) and patient-derived fibroblasts (*n* = 17 assays) (*P* [patient versus control] < 0.001; *P* [myriocin] < 0.01; *P* [etomoxir] = 0.103; *P* [interaction: myriocin-etomoxir] < 0.01) (*D*). Supplementation of etomoxir (gray boxes) prevents the increase of PINK1-deficient OCR following myriocin treatment. (*E*) ATP levels of Pink1-deficient flies that carry a heterozygous loss in CPT2 and that were supplemented with myriocin (blue dots) (*n* = 7 assays of two flies/assay) (*P* [pink1 versus control] < 0.01; *P* [myriocin] = 0.956; *P* [interaction] = 0.518). (*F*) OCR of Pink1-deficient flies heterozygous for CPT2 and that were supplemented with myriocin (blue dots) (*n* = 5 assays) (*P* [pink1 versus control] < 0.05; *P* [myriocin] = 0.610; *P* [interaction] = 0.865. Panels that do not present data from flies are labeled accordingly. Data are individual data points with means (*A*–*D*) or percentages (*E*); error bars: SEM. (*A* and *B*) Mann–Whitney *U* test; (*C*–*F*) nonparametric analyses of variances; ns: not significant; *P < 0.05; **P < 0.01; ***P < 0.001; pairwise comparisons as guidance can be found in the descriptive pairwise comparison of the supplemental information (*C*–*F*).

β-oxidation is the process in which fatty acids are broken down to generate acetyl-CoA that provides substrates for the cellular energy production. β-oxidation occurs in mitochondria and peroxisomes (33). Interestingly, ceramide accumulation negatively regulates β-oxidation (34). Hence, we investigated if increased β-oxidation, due to decreased ceramide levels, is responsible for the observed rescue. We indeed observed lower β-oxidation in *pink1*-mutant flies (Fig. 3*A*) and patient-derived fibroblasts (Fig. 3*B*) compared to control in flies and fibroblasts. Furthermore, ceramide accumulation adversely affects the ETC (24). Thus, to test if this effect plays a role in PINK1 deficiency, we analyzed the mitochondrial oxygen consumption rate (OCR) in isolated mitochondria from flies and in patient-derived fibroblasts using an extracellular oxygen probe. Supplementation of 300 μM myriocin resulted in increased OCR in PINK1-deficient flies (Fig. 3*C*) and fibroblasts (Fig. 3*D*). To assess if the improved phenotypes following myriocin treatment are the result of elevated β-oxidation, we inhibited β-oxidation via application of etomoxir, which lowers the import of fatty acids into mitochondria for β-oxidation by binding to carnitine palmitoyl transferase (CPT) (35). The observed rescue in the OCR with myriocin was absent when etomoxir was applied (Fig. 3 *C* and *D*, gray boxes). However, etomoxir supplementation had no effect on the OCR of *pink1*-mutant mitochondria and patient-derived fibroblasts that were not supplemented with myriocin (Fig. 3 *C* and *D*, gray boxes). To further validate these findings, we took advantage of flies with heterozygous loss of CPT2. Similarly, the rescuing effect in ATP levels and OCR with myriocin supplementation in *pink1*-mutant flies disappeared when β-oxidation was prevented due to heterozygous loss of CPT (Fig. 3 *E* and *F*). Thus, these data indicate that ceramide accumulation decreases β-oxidation in *PINK1* mutants, which can be rescued by lowering ceramide levels.

### Stimulation of β-Oxidation Rescues Loss of PINK1.

**Fig. 4.**
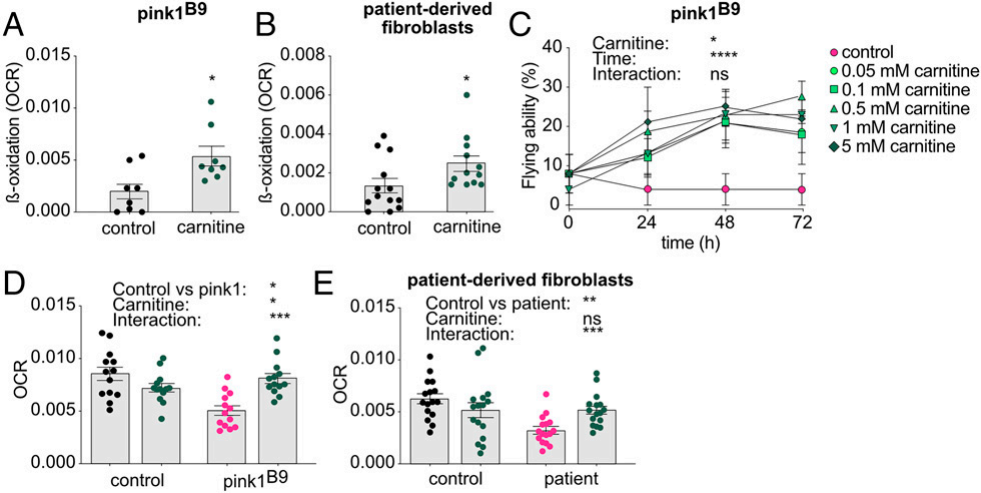
Stimulation of β-oxidation rescues loss of PINK1. (*A* and *B*) β-oxidation levels displayed as OCR in *pink1^B9^*-mutant flies (*n* = 8 assays) (*P* < 0.05) (*A*) and patient-derived fibroblasts (*n* = 12 to 13 assays) (*P* < 0.05) (*B*) that were treated with carnitine (5 mM for flies and 125 μM for fibroblasts) (green dots). (*C*–*E*) Supplementation of carnitine (green dots) increases flying ability (*n* > 50 flies) (*P* [carnitine] < 0.05; *P* [time] < 0.0001; *P* [interaction] = 0.3753) (*C*) and increases the OCR in flies (*n* = 13 assays) (*P* [pink1 versus control] < 0.05; *P* [carnitine] < 0.05; *P* [interaction] < 0.001) (*D*) and patient-derived fibroblasts (*n* = 16 assays) (*P* [patient versus control] < 0.01; *P* [carnitine] = 0.435; *P* [interaction] < 0.001) (*E*). Panels that do not present data from flies or that show data derived from *pink1*-mutant flies with and without drugs are labeled accordingly. Data are individual data points with means (*A* and *B*; *D* and *E*) or percentages (*C*); error bars: SEM. (*A* and *B*) Mann–Whitney *U* test; (*C*–*E*) nonparametric analysis of variances; ns: not significant; *P < 0.05; **P < 0.01; ***P < 0.001; ****P < 0.0001; pairwise comparisons as guidance can be found in the descriptive pairwise comparison of the supplemental information (*D* and *E*); circles depict the cell nucleus; arrowheads indicate the accumulations (mitochondria). (Scale bar, 5 μm.)

Carnitine-mediated import of fatty acids via the CPT is an essential step in β-oxidation (36). To test if carnitine can stimulate β-oxidation, we supplemented carnitine to the fly and cell medium, and our data imply an elevation of β-oxidation in PINK1-deficient flies and fibroblasts (Fig. 4 *A* and *B*). Next, we tested if stimulation of β-oxidation via carnitine supplementation can alleviate the observed phenotypes. An improvement was observed in the flying ability of *pink1^B9^*-mutant flies (Fig. 4*C*). Furthermore, stimulation of β-oxidation via 5 mM carnitine supplementation induced a partial improvement in ATP levels and mitochondrial morphology (*SI Appendix*, Fig. S3) and is sufficient to increase the OCR (Fig. 4*D*) upon loss of Pink1. Supplementation of 125 μM carnitine to the fibroblast medium to stimulate β-oxidation similarly resulted in improved OCR in *PINK1*-mutant patient-derived fibroblasts (Fig. 4*E*). Consequently, our data indicate that stimulation of β-oxidation is sufficient to improve the ETC and its downstream phenotypes following loss of PINK1 and thus can provide a therapeutic target in PINK1-related PD.

### Ceramide Accumulation Increases Autophagy.

**Fig. 5.**
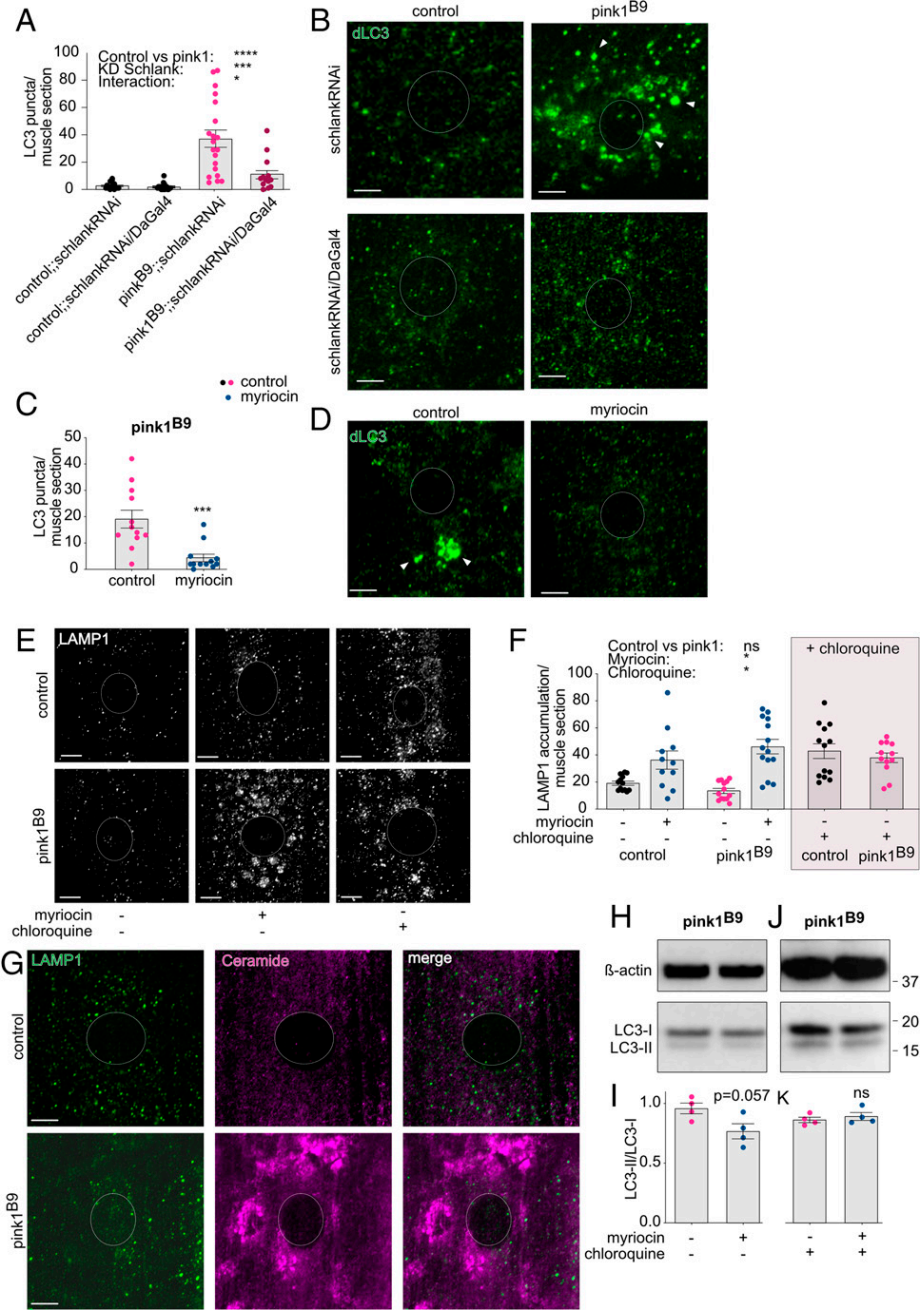
Ceramide accumulation increases autophagy. (*A*–*D*) Immunolabeling of third instar larval muscle sections visualizing dLC3 showed accumulations of dLC3 labeling (LC3 puncta ≥ 1.5 μm^2^) in Pink1-deficient flies that are absent in control flies (*n* = 20 muscle sections; four larvae) (*A*: quantification; *B*: images). This accumulated LC3 disappeared upon knockdown of Schlank (*P* [pink1 versus control] < 0.0001; *P* [DaGal4] < 0.001; *P* [interaction] < 0.05) (*A* and *B*) or 300 μM myriocin supplementation (blue dots) (*n* = 12 muscle sections; four larvae) (*P* < 0.001) (*C*: quantification; *D*: images). (*E*–*K*) Chloroquine (1 mg/mL) was applied to perform autophagy flux experiments. The 300 μM myriocin treatment (blue dots) inhibits autophagy flux similar to chloroquine (gray box) treatment as shown by increased LAMP1 accumulations (≥0.25 μm^2^) (*n* = 12 to 15 larval muscle sections) (*P* [pink1 versus control] = 0.642; *P* [chloroquine] < 0.05; *P* [interaction myriocin] < 0.05); (*E*: images; *F*: quantification). (*G*) Images of colabeling of LAMP1 and ceramide in control flies and *pink1*-mutant flies (quantification: *SI Appendix*, Fig. S5*G*). (*H* and *I*) Western blot analyses showed lower dLC3-II/dLC3-I levels in *pink1*-mutant flies treated with 300 μM myriocin (blue dots) compared to *pink1* mutants that were not treated with myriocin as a measure for lower autophagy flux (*n* = 4 assays) (*P* = 0.057) (*H*: images; *I*: quantification). Chloroquine dissipated the lower dLC3-II/dLC3-I levels following myriocin treatment (*n* = 4 assays) (*P* = 0.686) (*J*: images; *K*: quantification). Panels that show data derived from *pink1*-mutant flies with and without drugs are labeled accordingly. Data are individual data points with means; error bars: SEM. (*A*, *F*) nonparametric analysis of variances; (*C*) Mann–Whitney *U* test; ns: not significant; *P < 0.05; ***P < 0.001; ****P < 0.0001; pairwise comparisons as guidance can be found in the supplemental information (*A*, *F*); circles depict the cell nucleus; arrowheads indicate the accumulations (LC3). (Scale bar, 5 μm.)

In an attempt to understand the initial increase of ceramide upon PINK1 deficiency, we investigated whether this accumulation was a result of mitochondrial stress. To test this, we analyzed ceramide immunolabeling in flies with mitochondrial stress, such as knockdown of Cox15 and knockdown of CytC1. Our data show that mitochondrial stress increased ceramide accumulations compared to control flies (*SI Appendix*, Fig. S4). Thus, these data suggest that ceramide accumulates in response to mitochondrial stress. Defective mitochondria need to be cleared and, remarkably, autophagy can be triggered by ceramide (37). In addition, PINK1 functions in mitochondrial-specific autophagy (11), suggesting a potential link between ceramide accumulation and loss of PINK1. To assess possible effects on autophagy, we performed immunolabeling using antibodies against LC3, an autophagy marker. While immunolabeling of LC3 showed ubiquitous staining in control larvae, in *pink1*-mutant larvae, LC3 accumulated (Fig. 5 *A* and *B*), and accumulation disappeared upon (genetic or pharmacological) reduction of ceramide (Fig. 5 *A*–*D*). These data are further supported by Western blotting analyses that showed lowered LC3 levels following (genetic or pharmacological) reduction of ceramide upon Pink1 deficiency (*SI Appendix*, Fig. S5 *A*–*D*), suggesting that ceramide-triggered autophagy is activated in *pink1*-mutant flies. We observed a similar but less pronounced effect on LC3 accumulation in *park^1/Δ21^*-mutant larvae that were supplemented with myriocin (*SI Appendix*, Fig. S5 *E* and *F*). To investigate whether these defects are linked to altered autophagy flux, we applied chloroquine, a standard drug to reduce autophagy flux by inhibiting fusion of the autophagosome with the lysosome in the final steps of autophagy (38). This block in fusion as a result of chloroquine treatment leads to increased accumulation of LAMP1, a lysosomal marker (38). We confirmed the increased LAMP1 accumulation following chloroquine treatment in control flies (Fig. 5 *E* and *F*; chloroquine: gray box) and found that the amount of LAMP1 accumulation in *pink1* mutants is similar to those observed in control flies (Fig. 5 *E* and *F*), suggesting that LC3 levels in *pink1* mutants are increased in an attempt to maintain a basic level of autophagy flux. Interestingly, the supplementation of myriocin to the fly medium increased the LAMP1 accumulations in *pink1*-mutant flies (Fig. 5 *E* and *F*), mimicking the effect of chloroquine and thus suggesting that the reduction of ceramide lowers autophagy flux. Nonetheless, ceramide accumulation did not coincide with LAMP1 immunolabeling in Pink1 deficiency (Fig. 5*G* and *SI Appendix*, Fig. S5*G*). LC3-puncta, following myriocin supplementation, appeared not to be affected by chloroquine treatment (*SI Appendix*, Fig. S5 *H* and *I*). Nonetheless, Western blot analysis of the LC3-II/LC3-I ratio, a parameter for the maturation of the autophagosome, another measure for autophagy, tended to be lower following myriocin treatment (Fig. 5 *H* and *I*), while chloroquine abolished the effect of myriocin on the LC3-II/LC3-I ratio (Fig. 5 *J* and *K*). These data suggest that the ceramide accumulation induces autophagy initiation and autophagosome maturation in PINK1 deficiency. This might be a compensatory mechanism (of cells) to maintain a basic level of autophagy upon loss of PINK1, and thus, ceramide accumulates to increase the autophagy initiation to maintain a basic level of autophagy upon loss of PINK1.

### Ceramide Accumulation Coincides with Ceramide-Induced Mitophagy.

**Fig. 6.**
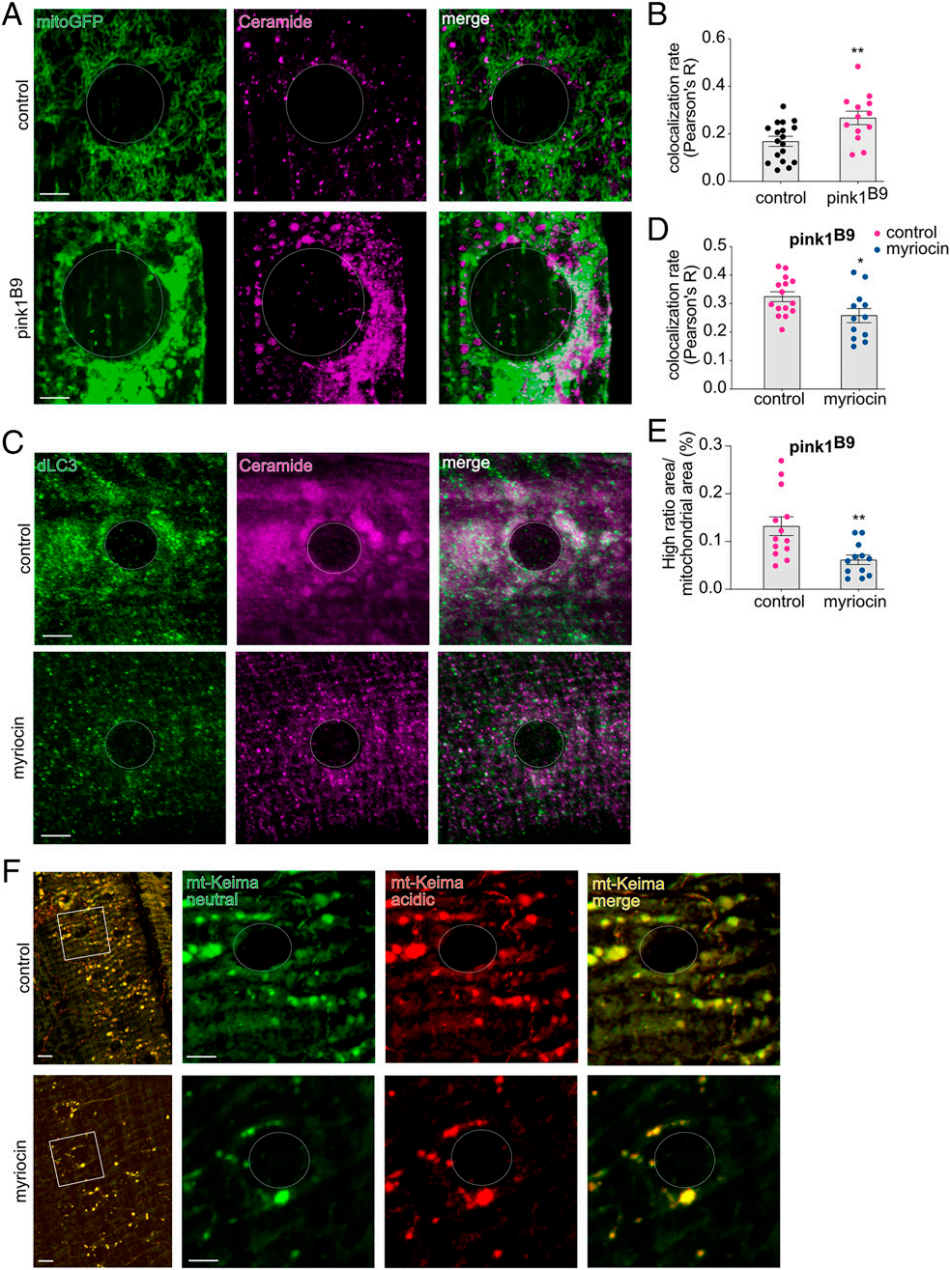
Ceramide accumulation coincides with ceramide-induced mitophagy. (*A* and *B*) Pink1-deficient flies displayed increased colocalization between mitochondria (mitoGFP) and ceramide, measured via the Pearson’s R value. (*n* = 13 to 18 muscle sections, 4 larvae) (*P* < 0.01) (*A*: images; *B*: quantification). (*C* and *D*) The increased colocalization, depicted via the Pearson’s R value, of dLC3 and ceramide was reduced following 300 μM myriocin treatment (blue dots) (*n* = 12 to 15 muscle sections, 4 larvae) (*P* < 0.05) (*C*: images; *D*: quantification). (*E* and *F*) Mt-Keima high ratio (acidic/neutral) area shows lower levels per mitochondrial area as a result of 300 μM myriocin treatment (blue dots) as an indication of reduced mitophagy (*n* = 12 to 13 muscle sections; 4 larvae) (*P* < 0.01) (*E*: quantification; *F*: images). Panels that show data derived from *pink1*-mutant flies with and without drugs are labeled accordingly. Data are individual data points with means; error bars: SEM. Mann–Whitney *U* test; *P < 0.05; **P < 0.01; circles depict the cell nucleus. (Scale bar, 5 μm.)

Colabeling of ceramide with mitochondria (expression of mitochondrial green fluorescent protein (mitoGFP) confirms the increased mitochondrial ceramide in Pink1 deficiency (Fig. 6 *A* and *B*), further corroborating our initial findings (Fig. 1 *A*–*F*). Interestingly, accumulation of mitochondrial ceramide serves as a signal for ceramide-induced mitophagy (21). In addition, PINK1 is involved in mitophagy (10, 11) and is indispensable for proper Complex I function (7). The inefficient functioning of Complex I upon loss of PINK1 impairs the ETC and, therefore, increases the number of defective mitochondria that fail to be cleared, resulting in mitochondrial aggregation and accumulation in *PINK1* mutants (6, 8, 9, 15). Hence, we hypothesized that the accumulation of ceramide is an attempt to facilitate mitochondrial clearance via ceramide-induced mitophagy. Lipidated LC3-II binds to accumulated mitochondrial ceramide to recruit the autophagosome for mitochondrial degradation (21, 39). The increased LC3 puncta and ceramide puncta observed in Pink1-deficient flies showed partial overlap (Fig. 6 *C* and *D*) that was decreased when ceramide levels were reduced following myriocin treatment (Fig. 6 *C* and *D*), suggesting that ceramide-induced mitophagy is activated upon Pink1 deficiency. To directly monitor mitophagy, we performed live imaging experiments using *pink1*-mutant flies that express the mt-Keima construct (40, 41), with and without the addition of myriocin to the fly medium. Basal mitophagy levels are initially maintained in *pink1*-mutant flies, but upon aging, mitophagy is impaired upon loss of Pink1 (40, 41), which is suggestive for the existence of an (initial) compensatory mechanism. The addition of myriocin to reduce ceramide diminished mitophagy levels (Fig. 6 *E* and *F*), suggesting that ceramide-induced mitophagy contributes to the basal mitophagy levels observed upon Pink1 deficiency.

## Discussion

**Fig. 7.**
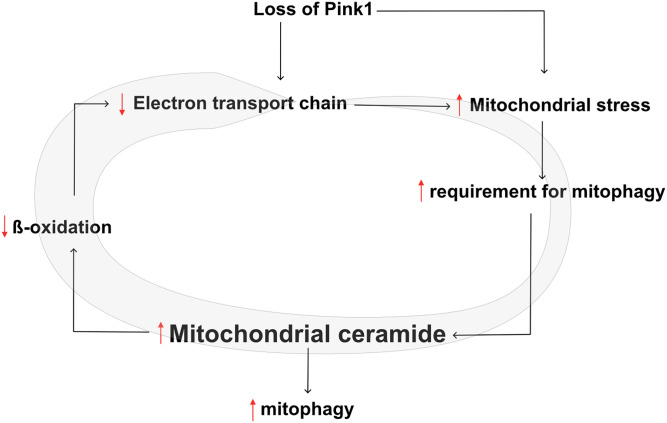
Simplified scheme of our proposed model in which loss of Pink1 results in a defective ETC followed by increased mitochondrial stress. This mitochondrial stress raises the necessity for mitochondrial clearance that is aided by the increase of mitochondrial ceramide to provoke ceramide-induced mitophagy. As a consequence, β-oxidation decreases, leading to a further decline of ETC efficiency, creating a vicious cycle that is indicated by the gray circular arrow that increases in size.

Lipids are undoubtedly connected to PD (5, 42, 43), but the underlying mechanisms are currently only incompletely understood. Here, we found accumulation of the sphingolipid ceramide at PINK1-deficient mitochondria to activate ceramide-induced mitophagy in an attempt to meet the increased requirements for mitochondrial clearance following reduced Complex I activity and mitochondrial dysfunction in PINK1 deficiency (Fig. 7).

Lipid aggregates are accompanied by mitochondrial damage as part of the Lewy body pathology in PD (5), pointing to a direct link between mitochondrial damage and disrupted lipid homeostasis in the disease pathogenesis. More specifically, disruption of sphingolipid homeostasis has previously been implicated in PD (42, 43). Elevated levels of ceramide, the primary sphingolipid from which all other sphingolipids are created, have been observed in several PD models (17, 44, 45), including in *PINK1* KO olfactory bulb in mice and in brains of an alpha-synuclein–overexpression fly model (17, 46). In patients, data on ceramide levels are conflicting. Increased ceramide levels in plasma have been reported in PD patients and, in addition, have been associated with cognitive impairment (19). In contrast, another study found lower ceramide levels and a shift to shorter ceramide species in postmortem brains of sporadic PD patients compared to controls (47). These discrepant data can be reconciled in light of the notion that the length of ceramide species defines their function (48). Furthermore, ceramide function is dependent on cellular localization, including the stabilization of the Golgi apparatus (49), fluidity of the endoplasmic reticulum (ER) membrane (50), and mitochondrial function (21, 24). Hence, to understand the effect of alterations in ceramide levels, it is important to assess the subcellular amount of ceramide. Our observations demonstrate increased ceramide levels in isolated mitochondria and altered ceramide distribution upon PINK1 deficiency. Remarkably, increased ceramide accumulations are sufficient to provoke mitochondrial defects, thus supporting the notion of accumulation of ceramide to exert a direct effect on mitochondrial function. In addition, our data further underline the importance of ceramide localization and distribution to define its function.

Interestingly, loss of PINK1 and mitochondrial ceramide accumulation both affect the mitochondrial ETC and mitophagy (6, 8–10, 21, 24, 32). Loss of PINK1 or ceramide accumulation result in impaired ETC function. In contrast, they have opposing effects on mitophagy: loss of PINK1 results in age-dependent defective mitophagy (41), while mitochondrial ceramide accumulation is a signal for ceramide-induced mitophagy (21).

Here, we showed that inhibition of ceramide synthesis improves *PINK1* mutant phenotypes, including ATP levels and ETC OCR in flies and fibroblasts, suggesting that the observed ceramide accumulation has an additive negative effect on the PINK1-deficient phenotypes. Despite this observation of ceramide accumulation worsening mitochondrial function, ceramide accumulation initially appears to be a consequence of mitochondrial stress. Previously, ceramide was shown to negatively correlate with β-oxidation (51, 52). In agreement with this notion, we discovered a decrease in β-oxidation in *PINK1* mutants due to ceramide accumulation. Furthermore, we found that the rescue in PINK1 deficiency following inhibition of ceramide synthesis disappears when β-oxidation is blocked. Conversely, our data revealed that stimulation of β-oxidation is sufficient to induce a rescue in PINK1-deficient flies and patient fibroblasts.

Functional PINK1 is required for mitophagy (32), and, consequently, the initially maintained basal levels of mitophagy upon PINK1 deficiency become impaired over time (40, 41). PINK1 stabilization at the mitochondrial membrane provides a signal for relocalization of Parkin to mitochondria to facilitate clearance of defective mitochondria (11, 32). Our data demonstrate mitochondrial accumulation of ceramide, ceramide-dependent accumulation of LC3, and increased colocalization of ceramide with LC3 in PINK1 deficiency, allowing a basal level of autophagy flux upon loss of PINK1. Previous reports show that mitophagy in *pink1* mutants is initially minimally affected (40, 41). However, mitophagy levels cannot be maintained upon aging (41), which is suggestive of a compensatory mechanism that fails to overcome the loss of PINK1-related mitophagy over time. Our data showing that reduction of ceramide lowers mitophagy supports the notion that ceramide-induced mitophagy aids in maintaining basal levels of mitophagy. However, this basal level of mitophagy cannot be maintained due to progressive ceramide accumulation over time, adding to the progressive loss of mitophagy levels in loss of Pink1. Furthermore, our data support a similar mechanism occurring in Parkin-deficient flies.

In conclusion, we propose a mechanism by which ceramide accumulates at the mitochondria to initiate mitophagy as an alternative mechanism to meet the increased requirements for mitochondrial clearance in PINK1 deficiency. However, ceramide accumulation is accompanied by counter effects such as lowered β-oxidation, which results in decreased ETC function and thus enhances *PINK1* mutant phenotypes, further adding to the necessity of mitochondrial clearance (Fig. 7). Under healthy conditions, neurons mostly produce energy via glucose metabolism (53); however, surrounding cells, such as glia cells, exclusively carry out β-oxidation in the brain. Glia cells play a central role in brain energy production when energy homeostasis is disrupted (54), as is observed in *PINK1* mutants. Interestingly, lipid production increases in activated neurons, forming a potential toxic hazard in these neurons that is scavenged by neighboring glia cells through their breakdown via β-oxidation. A lack of lipid uptake by glia cells results in neurodegeneration (55, 56). Our data further underline these findings, as the observed lowered β-oxidation due to ceramide accumulation contributes to the severity of the observed phenotypes that can be alleviated by stimulation of β-oxidation in PINK1 deficiency. Hence, we provide additional support toward stimulation of β-oxidation as a therapeutic intervention in neurodegeneration.

Thus, interfering with this vicious cycle by reducing ceramide levels or stimulating β-oxidation overcomes the energy deficit and, thus, may constitute a promising therapeutic target for the treatment of PD.

## Materials and Methods

Detailed procedures are provided in *SI Appendix, Detailed Materials and Methods*.

### Fly Genetics and Patient-Derived Fibroblasts

Fly genetics and human fibroblasts are listed in *SI Appendix, Detailed Materials and Methods*.

### Feeding Experiments.

Flies and fibroblasts were treated with myriocin and carnitine that were added to the standard molasses medium or cell culture medium. Detailed information is provided in *SI Appendix, Detailed Materials and Methods*.

### Flying Ability.

Flight assays were performed on 5-d-old male flies in batches of five flies each. Flies are placed in an empty vial, followed by gently tapping. Flies that were not able to fly were given a score of 0, while those that were able to fly were given a score of 1 (4). For feeding experiments, 1-d-old male flies were tested for flying ability and placed on the selected medium. Flying ability was performed every 24 h until 72 h.

### ATP.

ATP levels of adult flies (4) and patient-derived fibroblasts (16) were determined using the ATP Bioluminescence Assay kit CLS II (Roche) and a luminometer (Biotek). The values were normalized to total protein content that was measured using the bicinchoninic acid (BCA) method (Thermo Fisher). The entire procedure was performed repeatedly.

### OCR.

OCR was measured using the Extracellular Oxygen Consumption Assay (Abcam) following the manufacturer’s protocol. Detailed information is provided in *SI Appendix, Detailed Materials and Methods*.

### Immunolabeling and mt-Keima Imaging.

Third instar larval fillets were fixed for 20 min in 4% formaldehyde in phosphate buffered saline (PBS) and stained according to standard immunolabeling protocols as described in *SI Appendix, Detailed Materials and Methods*. Images were captured with an LSM710 confocal microscope and a 63× NA 1.4 oil lens and processed and analyzed as described in *SI Appendix, Detailed Materials and Methods*.

### Lipidomics.

Lipidomic analyses were performed on full homogenates and on isolated mitochondria by Lipotype Gesellschaft mit beschränkter Haftung (GmbH) as described (57). Further information is provided in *SI Appendix, Detailed Materials and Methods*.

### Statistics.

Experiments with sample sizes less than four per group were analyzed descriptively since statistical generalizations may in this case not be robust and the nonparametric tests we performed cannot reach statistical significance regardless of the effect size. For all other experiments, nonparametric analyses were performed using “R” version 4.0 with the packages rankFD and nparLD (*P < 0.05; **P < 0.01; ***P < 0.001). We conducted the following tests: Mann–Whitney *U* tests for the comparison of two independent groups; two-way and three-way nonparametric ANOVA for the effect of two or three factors and their interaction(s), respectively, on independent observations (58); and nonparametric ANOVA for longitudinal data for the effect of one factor in longitudinal data and interaction of the factor and time (59). Thus, for experiments with factorial designs, we do not compare groups in a univariate setting (i.e., test whether the shown groups are different), followed by pairwise comparisons. Instead, the factorial design of the experiments is taken into account as described in detail in the literature (58, 59). An example is given for Fig. 1*G*. One factor is the group (control versus pink1), the other is the knockdown (yes versus no). We can therefore test three effects, namely, 1) if the group has an effect independent of the knockdown; 2) if the knockdown has an effect independent of the group; and 3) if there is an interaction between these two factors (i.e., if the effect of the knockdown depends on the group) (Fig. 1*G*). Nonetheless, to facilitate further interpretation, however, descriptive pairwise comparisons have now been added to the descriptive pairwise comparison in the Supplement, although overall conclusions cannot be drawn from these *P* values. Individual data points are given; bar represents the mean ± SEM.

## Data Availability

All study data are included in the article and/or *SI Appendix*.
